# 5-HT_2_ receptor binding, functional activity and selectivity in *N*-benzyltryptamines

**DOI:** 10.1371/journal.pone.0209804

**Published:** 2019-01-10

**Authors:** Miguel Toro-Sazo, José Brea, María I. Loza, Marta Cimadevila, Bruce K. Cassels

**Affiliations:** 1 Department of Chemistry, Faculty of Sciences, University of Chile, Ñuñoa, Santiago, Chile; 2 BioFarma Research Group, CIMUS, Universidad de Santiago de Compostela, Santiago de Compostela, Spain; University of North Dakota, UNITED STATES

## Abstract

The last fifteen years have seen the emergence and overflow into the drug scene of “superpotent” *N*-benzylated phenethylamines belonging to the “NBOMe” series, accompanied by numerous research articles. Although *N-*benzyl substitution of 5-methoxytryptamine is known to increase its affinity and potency at 5-HT_2_ receptors associated with psychedelic activity, *N*-benzylated tryptamines have been studied much less than their phenethylamine analogs. To further our knowledge of the activity of *N*-benzyltryptamines, we have synthesized a family of tryptamine derivatives and, for comparison, a few 5-methoxytryptamine analogs with many different substitution patterns on the benzyl moiety, and subjected them to *in vitro* affinity and functional activity assays vs. the human 5-HT_2_ receptor subtypes. In the binding (radioligand displacement) studies some of these compounds exhibited only modest selectivity for either 5-HT_2A_ or 5-HT_2C_ receptors suggesting that a few of them, with affinities in the 10–100 nanomolar range for 5-HT_2A_ receptors, might presumably be psychedelic. Unexpectedly, their functional (calcium mobilization) assays reflected very different trends. All of these compounds proved to be 5-HT_2C_ receptor full agonists while most of them showed low efficacy at the 5-HT_2A_ subtype. Furthermore, several showed moderate-to-strong preferences for activation of the 5-HT_2C_ subtype at nanomolar concentrations. Thus, although some *N*-benzyltryptamines might be abuse-liable, others might represent new leads for the development of therapeutics for weight loss, erectile dysfunction, drug abuse, or schizophrenia.

## Introduction

Serotonin or 5-hydroxytryptamine (5-HT) is a bioactive compound present in a large variety of plants and animals. In mammals it is an autacoid or mediator of important functions in the gut and in blood platelets where it is most abundant, but in spite of its relative scarcity in the central nervous system its most widely known functions are as a neurotransmitter. The discovery of increasingly selective 5-HT receptor inhibitors has shown that serotonin is not only involved in important peripheral functions, but is also implicated in cognition, memory, emotion, the regulation of mood, the sleep-wake cycle, food intake, sexual activity, and in migraine, obsessive-compulsive disorder, schizophrenia and hallucinations [1]. Serotonin activates a large number of receptor subtypes (14 to date). With the exception of the 5-HT_3_ receptor which is a ligand-gated ion channel, serotonin receptors couple to G proteins, and are thus related to the release of second messengers such as cyclic adenosine, inositol phosphate(s) and arachidonic acid. However, signaling via β-arrestin recruitment is an important alternative signaling route that may be involved in different pharmacological outcomes [2].

The 5-HT_2_ receptors form a close-knit trio of G_q/11_ protein-coupled subtypes, with 5-HT_2A_ and 5-HT_2C_ showing somewhat greater sequence identity than the 5-HT_2B_ subtype but still with more than 50% overall sequence similarities [3]. The 5-HT_2A_ subtype is of particular relevance to schizophrenia and hallucination, and also seems to be involved in cognition, emotion, etc. The action of modern antipsychotic drugs such as clozapine and risperidone have a major 5-HT_2A_ antagonist component. In contrast, many full or partial 5-HT_2A_ agonists are well known hallucinogens, and classic psychedelics are believed to act primarily as 5-HT_2A_ receptor agonists [4,5]. Although the 5-HT_2B_ receptor is expressed in the central nervous system and drugs affecting its activity might be of therapeutic interest, it is now generally considered an antitarget due to the serious cardiovascular effects associated with its activation [6]. Finally, 5-HT_2C_ receptor agonists have attracted attention over the last decade as appetite suppressants and as possible agents for the treatment of drug abuse, erectile dysfunction, and schizophrenia [7–9]. Very recently, positive modulators have been identified as an alternative for increasing 5-HT_2C_ receptor signaling [10]. While a good number of 5-HT_2_ receptor subtype-selective antagonists have been identified, selective agonists are relatively rare and constitute an active field of research.

The investigation more than two decades ago of two series of *N*-benzyl and *N*-(4-substituted)benzyl derivatives of the psychedelic 4-bromo-2,5-dimethoxyphenethylamine (2C-B) and 5-methoxytryptamine suggested that these modifications induced mostly insignificant changes in 5-HT_2A_ receptor binding. Significant losses in affinity were observed with the 5-HT_2C_ receptor, leading to slight preferences for the 5-HT_2A_ subtype (in only some cases up to 10-fold or little more) [11]. In contrast, the finding that *N*-benzylation caused a 4 to 5-fold increase in potency of the weak partial agonist 3-aminoethyl-2,4-(1*H*,3*H*)-quinazolinedione at 5-HT_2A_ receptors, and that this effect was more marked with 2-methoxybenzyl substitution, led to the synthesis of a small set of tryptamine and phenethylamine derivatives [12–19], resulting in the discovery of the now notorious NBOMe drugs. A search in PubMed for the item “nbome” showed that at most two articles were published each year before 2010, the rate of publication rose to 25 by 2015, fell somewhat the next year, and then reached 32 in 2017, and 15 until mid-2018. Extensive structure-activity studies showed that most of the “superpotent” *N*-benzylated 2,5-dimethoxy-4-X-phenethylamines had negligible selectivity between 5-HT_2A_ and 5-HT_2C_ receptors [13,16–20]. This led to a quest for more 5-HT_2A_-selective agonists, which was successful in very few cases [15,16].

The older literature records limited exploration of *N*-benzyl and *N*-4-substituted benzyl-5-methoxytryptamines [11], and doctoral theses addressing *N*-2-hydroxy- or–methoxybenzyl derivatives of tryptamine and 5-methoxytryptamine [12,14,21]. The only recent, systematic study, is the paper by Nichols [19] showing for the first time that introduction of a *meta-*methoxyl, methylthio or methyl group, or a chlorine, bromine or iodine atom on the benzyl substituent, is equally effective in raising 5-HT_2A_ receptor affinities to low nanomolar *K*_i_ values, while *ortho-*methoxy or -bromo substitution are somewhat less favorable. Also, both agonist and antagonist radioligand displacement from the 5-HT_2A_ receptor is usually favored minimally (by a factor of 2 to 4) over the 5-HT_2C_ subtype. *In vitro* (Ca^2+^ mobilization) functional assays showed that almost all these compounds are high efficacy partial to full agonists at both receptor subtypes, in most cases with a tenfold or greater preference for the 5-HT_2A_ receptor, and up to 40-fold for the 3-iodobenzyl derivative.

**Fig 1:**
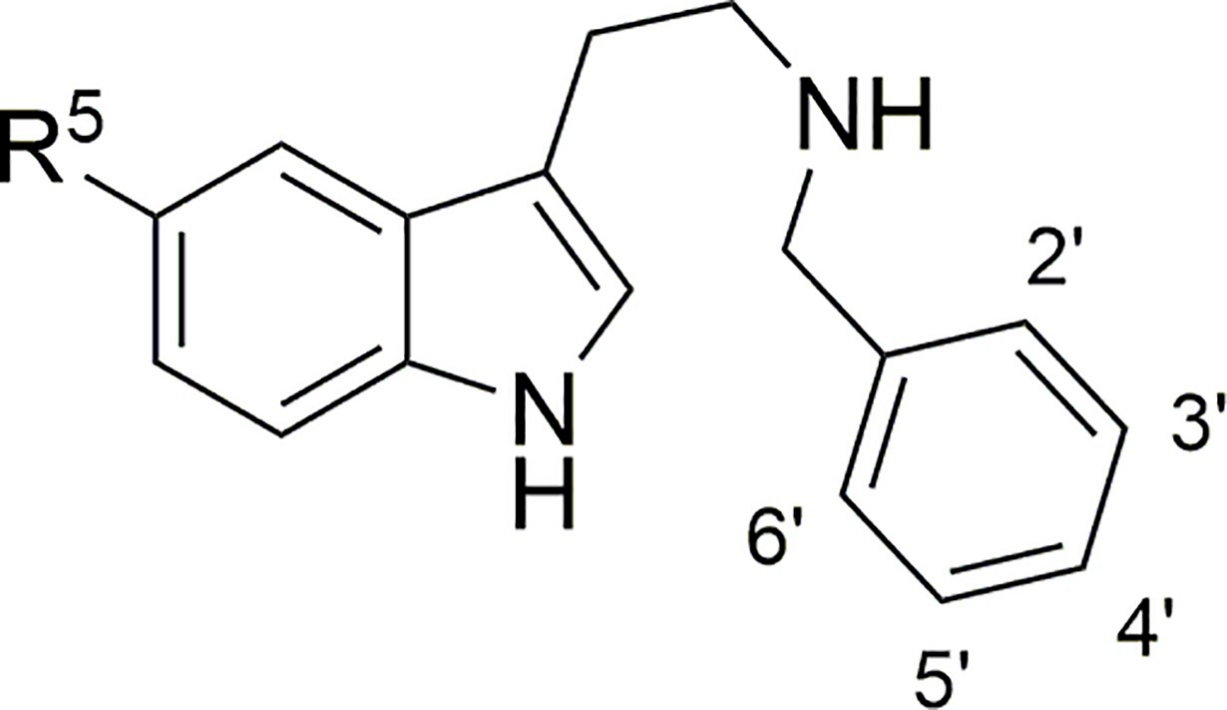
General structure and numbering of the *N*-benzyltryptamines. R^5^ = H, tryptamines; R^5^ = MeO, 5-methoxytryptamines.

The literature records only three *N-*benzylated tryptamine derivatives lacking the 5-methoxy substituent, comparing them with the corresponding 5-methoxytryptamines in a rat tail artery assay [12,14]. These compounds were partial agonists at the 5-HT_2A_ receptor, and were 2–4 times less potent than the 5-methoxy analogs, results that might be reasonably attributed to the absence of a hydrogen bond accepting methoxyl group on the indole moiety. It should be pointed out that the orthosteric binding site of 5-HT_2_ receptors contains serine, threonine and tyrosine residues that form hydrogen bonds with agonist and antagonist ligands [22,23]. It could be further conjectured that *N*-benzylated compounds with less interactions in the 5-HT_2A_ receptor’s orthosteric site might more clearly reveal effects due to unmapped interactions in the extended binding site described for the highly homologous 5-HT_2B_ and 5-HT_2C_ receptor crystal structures [22–24]. In fact Halberstadt [25], citing Braden *et al*. [13], points out that “compounds having low-to-moderate affinity tend to be the most sensitive to the (*N*-benzyl) substitution”. We therefore decided to use tryptamine instead of its 5-methoxy derivative as the starting point for the synthesis and evaluation of a more extensive series of *N*-benzyl compounds. Nevertheless, we also prepared and assayed a small number of 5-methoxytryptamine derivatives for comparison of our data with the literature, and to see if this substitution on the indole ring is responsible for any consistent changes in affinity or potency (Fig 1).

For specific substitution patterns, see Tables 1 and 2.

**Table 1:** Human 5-HT_2_ receptor subtype binding affinities (p*K*_i_ ± SEM, and *K*_i_ in parentheses) and 5-HT_2A/2C_ and (in parentheses) 5-HT_2C/2A_ selectivities of serotonin, tryptamine, and the synthesized compounds.

Compound	R^5^	R^X^	R^Y^	5-HT_2A_ p*K*_i_ ± S.E.M. (*K*_i_)	5-HT_2B_ p*K*_i_ ± S.E.M. (*K*_i_)	5-HT_2C_ p*K*_i_ ± S.E.M. (*K*_i_)	Selectivity 5-HT_2A/2C_ (5-HT_2C/2A_)
5-HT	OH			6.03	N.D.	N.D.	N.D.
Tryptamine	H			5.39 ± 0.25 (4073.80)	6.96 ± 0.06 (109.65)	7.02 ± 0.08 (95.50)	0.021 (46.7)
**1**	H	H	H	6.61 ± 0.11 (245.47)	7.00 ± 0.06 (100)	6.73 ± 0.06 (186.21)	0.75 (1.33)
**2**	H	2-OH	H	6.94 ± 0.20 (114.82)	7.17 ± 0.05 (67.61)	7.07 ± 0.10 (85.11)	0.73 (1.37)
**3**	H	2-OMe	H	7.05 ± 0.14 (89.13)	7.33 ± 0.11 (46.77)	6.65 ± 0.11 (223.87)	2.56 (0.39)
**4**	H	2-Me	H	48 ± 1*	6.47 ± 0.07 (338.84)	6.18 ± 0.05 (660.69)	N.D.
**5**	H	2-Cl	H	7.92 ± 0.12 (12.02)	7.63 ± 0.04 (23.44)	7.61 ± 0.03 (24.55)	2.04 (0.49)
**6**	H	2-Br	H	6.71 ± 0.08 (194.98)	7.13 ± 0.07 (74.13)	6.47 ± 0.12 (338.84)	1.74 (0.57)
**7**	H	3-OH	H	7.12 ± 0.07 (75.86)	7.43 ± 0.07 (37.15)	7.59 ± 0.06 (25.70)	0.36 (2.8)
**8**	H	3-Me	H	7.84 ± 0.06 (14.45)	7.77 ± 0.03 (16.98)	7.13 ± 0.03 (74.13)	5.10 (0.20)
**9**	H	3-F	H	6.59 ± 0.08 (257.04)	6.90 ± 0.05 (125.89)	6.67 ± 0.06 (213.80)	0.84 (1.19)
**10**	H	3-Cl	H	7.35 ± 0.07 (44.67)	7.46 ± 0.06 (34.67)	7.01 ±0.08 (97.72)	2.17 (0.46)
**11**	H	3-Br	H	8.09 ± 0.14 (8.13)	7.66 ± 0.07 (21.88)	7.12 ± 0.07 (75.86)	8 (0.13)
**12**	H	4-OH	H	6.04 ± 0.12 (912.01)	6.31 ± 0.08 (489.78)	6.00 ± 0.08 (1000)	1.09 (0.92)
**13**	H	4-OMe	H	6.34 ± 0.10 (457.09)	7.16 ± 0.10 (69.18)	6.45 ± 0.08 (354.81)	0.78 (1.28)
**14**	H	4-Me	H	6.38 ± 0.08 (416.87)	7.13 ± 0.04 (74.13)	6.48 ± 0.04 (331.13)	0.81 (1.23)
**15**	H	4-OEt	H	6.56 ± 0.09 (275.42)	6.57 ± 0.06 (269.15)	6.13 ± 0.11 (741.31)	2.66 (0.36)
**16**	H	4-Cl	H	6.15 ± 0.10 (707.95)	6.65 ± 0.14 (223.87)	6.02 ± 0.08 (954.99)	1.37 (0.73)
**17**	H	4-Br	H	6.00 ± 0.06 (1000)	6.58 ± 0.09 (263.03)	5.97 ± 0.08 (1071.52)	1.09 (0.92)
**18**	H	4-NO_2_	H	5.58 ± 0.07 (2630.27)	6.70 ± 0.11 (199.53)	5.85 ± 0.11 (1412.54)	0.54 (1.85)
**19**	H	2-OH	3-OMe	7.58 ± 0.06 (26.30)	7.88 ± 0.06 (13.18)	7.78 ± 0.06 (16.60)	0.58 (1.72)
**20**	H	2-OMe	3-OMe	5.82 ± 0.16 (1513.56)	6.71 ± 0.03 (194.98)	5.95 ± 0.07 (1122.02)	0.73 (1.37)
**21**	H	2-OH	3-Br	7.85 ± 0.05 (14.13)	7.81 ± 0.07 (15.49)	6.86 ± 0.08 (138.04)	9.8 (0.10)
**22**	H	2-OH	3-F	6.68 ± 0.05 (208.93)	6.89 ± 0.04 (128.82)	6.75 ± 0.07 (177.83)	0.82 (1.22)
**23**	H	2-OH	5-Me	6.13 ± 0.06 (741.31)	6.81 ± 0.04 (154.88)	6.57 ± 0.08 (269.15)	0.36 (2.77)
**24**	H	2-OH	5-F	6.12 ± 0.04 (758.58)	7.11 ± 0.07 (77.62)	6.98 ± 0.07 (104.71)	0.14 (7.15)
**25**	H	2-OMe	5-F	6.44 ± 0.08 (363.08)	7.02 ± 0.07 (95.50)	6.82 ± 0.14 (151.36)	0.42 (2.40)
**26**	H	2-OH	5-Br	6.51 ± 0.09 (309.03)	N.D.	6.14 ± 0.06 (724.44)	2.39 (0.42)
**27**	H	2-OMe	5-Br	5.95 ± 0.09 (1122.02)	7.04 ± 0.05 (91.20)	6.87 ± 0.09 (134.90)	0.12 (8.22)
**28**	H	2-OMe	5-Cl	6.01 ± 0.09 (977.24)	6.10 ± 0.06 (794.33)	5.88 ± 0.06 (1318.26)	1.35 (0.74)
**29**	H	2-OMe	5-OMe	6.48 ± 0.09 (331.13)	7.24 ± 0.04 (57.54)	7.06 ± 0.06 (87.10)	0.26 (3.85)
**30**	H	2-OH	5-NO_2_	8 ± 4*	6.05 (891.25)	31 ± 4*	N.D.
**31**	H	2-OH	4-Br	5.81 ± 0.10 (1548.82)	6.96 ± 0.04 (109.65)	6.22 ± 0.05 (602.56)	0.38 (2.63)
**32**	H	2-OMe	4-OMe	6.18 ± 0.09 (660.69)	6.63 ± 0.04 (234.42)	6.57 ± 0.09 (269.15)	0.41 (2.44)
**33**	H	2-OH	6-Br	5.78 ± 0.05 (1659.59)	7.16 ± 0.02 (69.18)	6.95 ± 0.09 (112.20)	0.067 (15)
**34**	H	2-OH	6-F	6.64 ± 0.06 (229.09)	6.94 ± 0.04 (114.82)	6.81 ± 0.08 (154.88)	0.95 (1.05)
**35**	H	2-OH	3,5-diBr	12 ± 4*	5.22 (6025.60)	26 ± 4*	N.D.
**36**	H	3-OMe	4-OMe	5.89 ± 0.10 (1288.25)	6.54 ± 0.07 (288.40)	5.92 ± 0.11 (1202.26)	0.92 (1.09)
**37**	OMe	H	H	7.48 ± 0.07 (33.11)	7.78 ± 0.11 (16.60)	7.02 ± 0.05 (95.50)	2.93 (0.34)
**38**	OMe	2-OMe	H	7.35 ± 0.05 (44.67)	7.80 ± 0.06 (15.85)	7.16 ± 0.07 (69.18)	1.55 (0.65)
**39**	OMe	2-Cl	H	7.87 ± 0.06 (13.49)	7.43 ± 0.09 (37.15)	7.13 ± 0.08 (74.13)	5.51 (0.18)
**40**	OMe	2-Br	H	7.91 ± 0.09 (12.30)	7.54 ± 0.05 (28.84)	7.15 ± 0.07 (70.79)	5.66 (0.18)
**41**	OMe	4-Br	H	6.41 ± 0.04 (389.05)	6.81 ± 0.07 (154.88)	6.42 ± 0.04 (380.19)	0.98 (1.02)
**42**	OMe	2-OH	5-OMe	6.87 ± 0.04 (134.90)	7.69 ± 0.05 (20.42)	7.39 ± 0.07 (40.74)	0.30 (3.31)
**43**	OMe	2-OH	5-F	8.40 ± 0.16 (3.98)	8.05 ± 0.07 (8.91)	7.40 ± 0.09 (39.81)	9.66 (0.10)

*Binding inhibition at 10 μM

**Table 2:** Human 5-HT_2_ receptor subtype Ca^2+^ mobilization potencies (p*EC*_50_ ± SEM, and *EC*_50_ in parentheses) and relative efficacies (% of response to 5-HT), and 5-HT_2A/2C_ and (in parentheses) 5-HT_2C/2A_ selectivities of serotonin, tryptamine, and the synthesized compounds.

Compound	R^5^	R^X^	R^Y^	5-HT_2A_		5-HT_2C_		Selectivity
				p*EC*_50_	% *E*_max_	p*EC*_50_	% *E*_max_	5-HT_2A/2C_ (5-HT_2C/2A_)
5-HT	OH	—	—	8.09 ± 0.06 (8.13)	99.72 ± 1.93	9.87 ± 0.07 (0.13)	98.29 ± 2.03	0.016 (62.5)
Tryptamine	H	—	—	7.76 ± 0.07 (17.38)	97.60 ± 2.34	8.93 ± 0.13 (1.17)	107.8 ± 3.43	0.067 (14.9)
**1**	H	H	H	6.79 ± 0.24 (162.18)	61.65 ± 5.17	7.30 ± 0.15 (50.12)	121.4 ± 6.58	0.31 (3.24)
**2**	H	2-OH	H	6.70 ± 0.11 (199.53)	94.13 ± 4.59	7.47 ± 0.12 (33.88)	85.85 ± 2.83	0.17 (5.89)
**3**	H	2-OMe	H	5.81 ± 0.07 (1548.82)	62.98 ± 1.95	7.45 ± 0.10 (35.48)	94.24 ± 3.75	0.023 (43.7)
**4**	H	2-Me	H	5.7 ± 0.15 (1995.26)	44.44 ± 2.79	6.86 ± 0.09 (138.04)	106.9 ± 2.90	0.069 (14.5)
**5**	H	2-Cl	H	6.33 ± 0.10 (467.74)	25.52 ± 1.16	6.71 ± 0.14 (194.98)	87.07 ± 4.96	0.42 (2.40)
**6**	H	2-Br	H	5.65 ± 0.27 (2238.72)	29.33 ± 5.93	6.63 ± 0.13 (234.42)	108.6 ± 5.20	0.10 (9.55)
**7**	H	3-OH	H	7.25 ± 0.23 (56.23)	52.75 ± 4.63	8.17 ± 0.20 (6.76)	96.34 ± 4.02	0.12 (8.32)
**8**	H	3-Me	H	7.21 ± 0.18 (61.66)	32.98 ± 2.11	7.53 ± 0.06 (29.51)	82.61 ± 2.11	0.48 (2.09)
**9**	H	3-F	H	6.41 ± 0.26 (389.05)	33.82 ± 4.01	6.95 ± 0.10 (112.20)	112.1 ± 4.84	0.29 (3.47)
**10**	H	3-Cl	H	6.92 ± 0.2 (120.23)	57.91 ± 4.15	6.55 ± 0.11 (281.84)	117.3 ± 4.82	2.34 (0.43)
**11**	H	3-Br	H	7.23 ± 0.14 (58.88)	32.59 ± 1.64	7.26 ± 0.07 (54.95)	106.7 ± 2.78	0.93 (1.07)
**12**	H	4-OH	H	N.D.	N.D.	6.84 ± 0.10 (144.54)	99.65 ± 2.81	N.D.
**13**	H	4-OMe	H	7.34 ± 0.06 (45.71)	108.2 ± 2.78	8.08 ± 0.06 (8.32)	91.44 ± 2.03	0.18 (5.49)
**14**	H	4-Me	H	N.D.	N.D.	5.92 ± 0.14 (1202.26)	100.5 ± 5.36	N.D.
**15**	H	4-OEt	H	6.44 ± 0.34 (363.08)	36.73 ± 6.13	7.93 ± 0.17 (11.75)	93.15 ± 3.26	0.032 (30.9)
**16**	H	4-Cl	H	7.23 ± 0.15 (58.88)	79.96 ± 4.54	7.30 ± 0.11 (50.12)	98.34 ± 4.18	0.85 (1.17)
**17**	H	4-Br	H	N.D.	N.D.	5.17 ± 0.08 (6760.83)	110.2 ± 4.30	N.D.
**18**	H	4-NO_2_	H	N.D.	N.D.	6.28 ± 0.19 (524.81)	92.05 ± 8.69	N.D.
**19**	H	2-OH	3-OMe	7.31 ± 0.34 (48.98)	26.16 ± 2.34	7.03 ± 0.15 (93.33)	107.4 ± 6.91	1.91 (0.52)
**20**	H	2-OMe	3-OMe	N.D.	N.D.	5.44 ± 0.26 (3630.78)	117.3 ± 9.18	N.D.
**21**	H	2-OH	3-Br	4.80 ± 0.24 (15848.93)	40.38 ± 6.23	7.56 ± 0.10 (27.54)	98.83 ± 2.83	0.0017 (575)
**22**	H	2-OH	3-F	6.79 ± 0.18 (162.18)	53.62 ± 4.49	7.77 ± 0.15 (16.98)	103.5 ± 4.96	0.105 (9.55)
**23**	H	2-OH	5-Me	6.96 ± 0.06 (109.65)	43.57 ± 0.90	7.00 ± 0.08 (100)	89.38 ± 3.86	0.91 (1.10)
**24**	H	2-OH	5-F	7.12 ± 0.09 (75.86)	71.64 ± 1.95	7.58 ± 0.12 (26.30)	105.2 ± 2.19	0.35 (2.88)
**25**	H	2-OMe	5-F	7.22 ± 0.09 (60.26)	57.98 ± 1.95	7.28 ± 0.12 (52.48)	109.7 ± 2.19	0.87 (1.15)
**26**	H	2-OH	5-Br	N.D.	N.D.	6.15 ± 0.08 (707.95)	107.8 ± 2.88	N.D.
**27**	H	2-OMe	5-Br	4.75 ± 0.08 (17782.79)	69.04 ± 3.51	6.58 ± 0.14 (263.03)	110.8 ± 4.63	0.015 (67.6)
**28**	H	2-OMe	5-Cl	5.05 ± 0.07 (8912.51)	69.21 ± 2.67	6.62 ± 0.10 (239.88)	109.7 ± 3.35	0.027 (37.2)
**29**	H	2-OMe	5-OMe	5.46 ± 0.04 (3467.37)	38.97 ± 0.67	6.88 ± 0.09 (131.83)	118.4 ± 4.45	0.038 (26.3)
**31**	H	2-OH	4-Br	4.61 ± 0.21 (24547.09)	62.53 ± 10.79	6.09 ± 0.09 (812.83)	74.75± 2.60	0.033 (30.2)
**32**	H	2-OMe	4-OMe	N.D.	N.D.	6.62 ± 0.13 (239.88)	117.2 ± 4.59	N.D.
**33**	H	2-OH	6-Br	5.23 ± 0.67 (5888.44)	34.40 ± 1.37	6.10 ± 0.08 (794.33)	103.5 ± 2.91	0.13 (7.41)
**34**	H	2-OH	6-F	7.40 ± 0.16 (39.81)	49.28 ± 3.23	7.73 ± 0.19 (18.62)	91.67 ±6.60	0.47 (2.13)
**36**	H	3-OMe	4-OMe	5.65 ± 0.35 (2238.7)	50.52 ± 8.27	7.24 ± 0.11 (57.54)	100.5 ± 2.85	0.026 (38.9)
**37**	OMe	H	H	7.69 ± 0.10 (20.42)	63.19 ± 1.44	7.84 ± 0.13 (14.45)	112.7 ± 3.47	0.71 (1.41)
**38**	OMe	2-OMe	H	8.70 ± 0.20 (1.99)	83.66 ± 3.42	8.42 ± 0.16 (3.80)	88.69 ± 4.22	1.91 (0.53)
**39**	OMe	2-Cl	H	7.92± 0.11 (12.02)	47.74 ± 1.57	7.09 ± 0.09 (81.28)	106.2 ± 4.32	6.76 (0.15)
**40**	OMe	2-Br	H	7.53 ± 0.06 (29.51)	44.79 ± 0.99	7.56 ± 0.14 (27.54)	105.1± 4.32	0.93 (1.07)
**41**	OMe	4-Br	H	5.73 ± 0.10 (1862.09)	60.53 ± 2.41	6.88 ± 0.15 (131.83)	93.52 ± 3.05	0.071 (14.1)
**42**	OMe	2-OH	5-OMe	6.96 ± 0.06 (109.65)	43.57 ± 0.90	7.00 ± 0.08 (100)	89.38 ± 3.86	0.91 (1.1)
**43**	OMe	2-OH	5-F	8.86 ± 0.10 (1.38)	83.26 ± 2.26	8.44 ± 0.12 (3.63)	106.1 ± 3.05	2.63 (0.38)

## Results and discussion

### Chemistry

**Fig 2:**
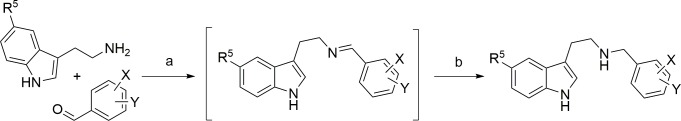
Synthesis of *N*-(substituted)benzyltryptamines. a: MeOH, r.t., overnight; b: NaBH_4_ in small portions, r.t.

All the compounds (thirty-six *N*-benzylated tryptamine derivatives and seven *N*-benzylated 5-methoxytryptamine derivatives, four of the latter described previously [11,19], were synthesized by treating appropriate benzaldehydes with tryptamine or 5-methoxytryptamine (free base) in methanol and reducing the unisolated imine intermediate with sodium borohydride, as described for phenethylamines and 5-methoxytryptamines and shown in Fig 2 [16,19,26]:

The secondary amines thus obtained in good yields, and not requiring extensive purification, were used as their water-soluble salts (usually hydrochlorides) in receptor binding and functional pharmacological studies.

**Fig 3:**
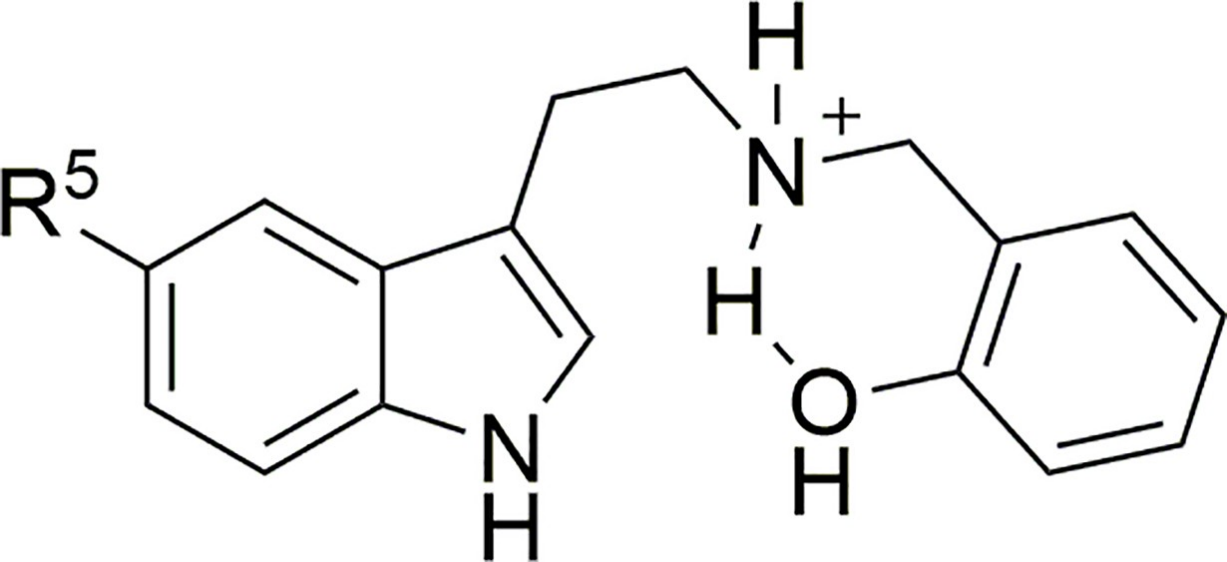
Intramolecular hydrogen bond in *N*-(2’-hydroxybenzyl)tryptamines.

It is worth noting that the ^1^H NMR spectra (in DMSO-*d*_6_) of some salts with an *ortho*-hydroxyl group on the *N*-benzyl moiety indicate the presence of an intramolecular hydrogen bond between the protonated amine and the hydroxyl group (Fig 3).

This steric constraint may help to understand differences in binding affinities and functional activities. A similar bond does not seem to be present in the *ortho*-methoxybenzylated compounds, possibly disfavored by the bulk of the *O*-methyl group.

### Pharmacology

#### Binding assays

The affinities of the products for human 5-HT_2A_, 5-HT_2B_, and 5-HT_2C_ receptors were evaluated by radioligand displacement from cultured cells expressing the appropriate human receptors (CHO h5-HT_2A_, CHO h5-HT_2B_, HeLa h5-HT_2C_). [^3^H]Ketanserin was used for 5-HT_2A_, [^3^H]LSD for 5-HT_2B_, and [^3^H]mesulergine for 5-HT_2C_ (Table 1).

It should be noted that the affinities at 5-HT_2A_ and 5-HT_2C_ receptors were determined by displacement of antagonist radioligands, and therefore reflect binding to both active and inactive receptor conformations, while an agonist, presumably binding to an active conformation, was used for the 5-HT_2B_ affinity determinations. Therefore, the 5-HT_2B_ affinities are not strictly comparable to the others, and were not considered in the selectivity estimates.

The vast majority of the our products revealed submicromolar affinities at all three receptor subtypes, and a small number gave *K*_i_ values in the 10 nanomolar range, although these values did not seem to follow any general trend. *N*-benzyltryptamine (**1**) bound to the 5-HT_2A_ and 5-HT_2C_ receptors with rather similar affinities to those reported for *N*,*N*-dimethyltryptamine (237 and 424 nM, respectively), but significantly better than *N*,*N*-diisopropyltryptamine (1.2 and 6.5 μM, respectively) [27]. *N*-Benzyl-5-methoxytryptamine (**37**) bound to both receptors 5–15 times more strongly than *N*-isopropyl-*N*-methyl-5-methoxytryptamine (165 nM and 1.3 μM, respectively) [27].

Comparison of our tryptamine derivatives with the corresponding 5-methoxylated analogs seemed to indicate some degree of parallelism. Thus, compounds **38**, **40**, and **41**, described recently [19], which in our hands gave similar binding results to those published, are the 5-methoxytryptamine analogs of the 2-methoxy-, 2-bromo- and 4-bromobenzyltryptamines **3**, **6**, and **17**, respectively. While in our case the human receptors were expressed in CHO (Chinese hamster ovary) cells, and in the paper by Nichols et al. [19] in human embryonic kidney (HEK) cells, our results for compounds **40** and **41** agree with theirs within a factor of 2. In the case of **38** our results do not differ by more than 2.7 times, which seems reasonable for data from different laboratories and determined in different biological substrates.

Compounds **3** and **17**, lacking the indole 5-methoxy group showed 2–4 times lower affinities than their indole-methoxylated counterparts **38** and **41**, at all three receptor subtypes, in line with the observations of Heim [12] and Silva *et al*. [14] for **1** vs. **37**. Contrary to expectations, the rather strongly binding *N*-(2-chlorobenzyl)tryptamine (**5**) had slightly greater affinity than its 5-methoxytryptamine analog **39**, at least at the 5-HT_2B_ and 5-HT_2C_ receptors. (Fig 1). It may be pointed out that, as noted by Jensen [21] and Nichols [19], the supposedly very high-affinity [11] *N*-4-bromobenzyl-5-methoxytryptamine (**41**) was not at all exceptional.

Compounds **8**, **10** and **11**, bearing a single hydrophobic substituent at the *meta* position of the benzyl group (CH_3_, Cl, or Br), bound rather strongly to the 5-HT_2_ receptors with 2 to 8-fold 5-HT_2A/2C_ selectivity, as had been seen for their 5-methoxytryptamine counterparts (respectively **5j**, **5h** and **5e** in that paper) [19]. Intriguingly, however, the 3-chlorobenzyl derivative **10** had somewhat lower affinity than the 2-chloro analog **5**. In contrast, 5-fluoro-2-hydroxybenzyl substitution gave profoundly different results in the tryptamine and the 5-methoxytryptamine series: the 5-methoxytryptamine derivative (**43**) had the highest 5-HT_2A_ affinity (p*K*_i_ = 8.4) of the whole series, while *N*-(5-fluoro-2-hydroxybenzyl)tryptamine (**24**) bound quite poorly to this receptor (p*K*_i_ = 6.1), and was slightly selective for the 2C subtype.

Almost half of our compounds bear an *ortho-*oxygen substituent on the benzyl moiety, which was introduced as a test of Heim’s “structure-activity concept” regarding the increased activity of 2-methoxybenzyl derivatives [12]. Comparing the *N-*benzyl- (**1**), *N-*2-hydroxybenzyl- (**2**) and *N-*2-methoxybenzyl- (**3**) tryptamines, 5-HT_2A_ (and also 5-HT_2B_) affinity appeared to increase slightly in that order, although at most by a factor of 2.7. At the 5-HT_2C_ receptor the affinities fluctuated in the same range. These changes can hardly be considered significant. Similarly, in several pairs of compounds in which binding could be compared, introduction of an *ortho* hydroxyl or methoxyl group had at most a very minor effect. The only exceptions were the *N-*3-chlorobenzyl- (**10**) and *N-*3-bromobenzyl- (**11**) tryptamines, where substitution to afford the *N-*5-chloro-2-methoxybenzyl (**28**) and *N-*5-bromo-2-methoxybenzyl (**11**) and to a lesser extent *N-*5-bromo-2-hydroxybenzyl (**26**) analogs, was markedly damaging, at least for 5-HT_2A_ receptor binding. Thus, the presence of an *ortho-*oxygen substituent on the benzyl ring does not seem to be generally beneficial and, with the exceptions of the 2-hydroxy-3-methoxybenzyl and the 2-hydroxy-3-bromobenzyl derivatives **19** and **21**, the *N*-disubstituted benzyltryptamines showed rather low affinities. On the other hand, in our limited 5-methoxytryptamine series, the 2-hydroxy-5-fluoro derivative **43** showed surprisingly strong 5-HT_2A_ receptor binding, and it also bound fairly strongly to the 5-HT_2C_ receptor.

#### Functional assays

The functional activities of our compounds at human 5-HT_2A_ and 5-HT_2C_ receptors were determined fluorometrically as calcium mobilization in CHO h5-HT_2A_ and HeLa h5-HT_2C_ cells (Table 2).

Comparison of our results for the *N-*2-methoxybenzyl- (**38**), *N-*2-bromobenzyl- (**40**), and *N-*4-bromobenzyl- (**41**) 5-methoxytryptamine derivatives with those of Nichols *et al*. [19] indicates complete agreement in functional potencies in one case, and differences of somewhat more than an order of magnitude in the others, although practically full agonism at the 5-HT_2C_ receptor seems to be the norm. The use of different cell lines is probably responsible in part for these discrepancies, but it must be kept in mind that 5-HT_2_ receptors display differential functional selectivity [28,29] and β-arrestin signaling bias [24] which are other factors to be considered.

Comparing tryptamine with 5-methoxytryptamine derivatives, the *N-*2-methoxybenzyl (NBOMe) derivative **3** was several hundred times less potent than its 5-methoxytryptamine analog **38** in the h5-HT_2A_ receptor functional assay, and the *N-*2-chlorobenzyl **5** was almost 40 times less potent than its counterpart **39**. A comparison of **17** with **41** was not possible because the functional activity of the former was too low for quantification. At the h5-HT_2C_ receptor, **3** and **5** were only 2–9 times less potent than the corresponding 5-methoxytryptamine derivatives. It seems likely that while a 5-methoxyl group on the indole moiety can result in considerably higher potency at the h5-HT_2A_ subtype, it may have a less significant effect at the h5-HT_2C_ receptor.

Almost two decades ago we found that the electrophysiologically determined rank potencies of several hallucinogenic and non-hallucinogenic phenylisopropylamines were consistent with the 5-HT_2A_ and 5-HT_2C_ affinities obtained in radioligand displacement assays, and the 2C/2A affinity ratios paralleled the potency ratios reported in that work [30]. Although both the voltage-clamp assay used then and the fluorescence assay used in the present work depend on intracellular calcium release, other mechanisms are active in cells and the final result of receptor activation may not show such correlations. As far back as 1997 a modified ternary receptor model was invoked to explain discrepancies [31], and it has recently been shown that the binding profile alone can reasonably predict strong hallucinogenic effects *in vivo* [18,32].

**Fig 4:**
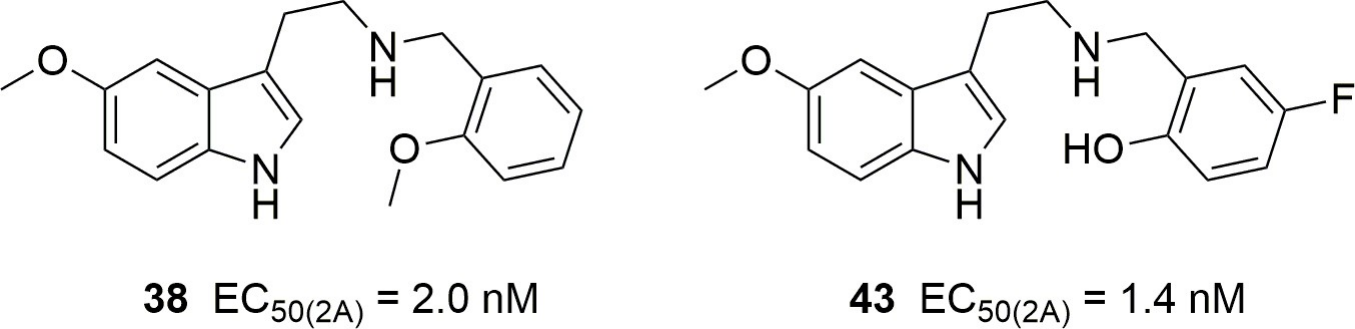
Possibly psychedelic *N*-benzyl-5-methoxytryptamines.

As seen with respect to the affinities of these compounds for the three receptor subtypes, apparently similar molecules sometimes behave quite differently, defying interpretation. Many of these substances seem uninteresting as 5-HT_2_ agonists because of their low potencies. However, a few of them exhibit low nanomolar *EC*_50_ values, at either subtype, without following any obvious rule. Interest in *N-*benzylated phenethylamines and indoleamines has focused mainly on their possible psychedelic activities related to full or partial agonism at 5-HT_2A_ receptors [4]. In this regard, it is worth noting that the reported 5-HT_2A_ affinities of *N-*benzyl-5-methoxytryptamines were generally several times lower than those of the correspondingly *N-*benzylated 2-(2,5-dimethoxy-4-iodophenyl)ethylamines, with the sole exception (30x) of the “superpotent” *N-*(2-methoxybenzyl) analog (25I-NBOMe), but their *in vitro* functional potencies did not follow this trend [19]. The rodent head twitch response is commonly believed to distinguish 5-HT_2A_ agonists that are psychedelic in humans from others that are not [25]. The *EC*_50_ values determined for those *N-*benzyl-5-methoxytryptamines which elicited the response (not all did) indicated potencies at least 30 times lower than that of 25I-NBOMe [19]. One could therefore expect that very few (e.g. **38** and **43**, Fig 3) of these compounds might be human hallucinogens in the low milligram dose range, significantly higher than the commonly abused NBOMe phenethylamines. Considering the binding affinities instead of the calcium mobilization data, a fair number of our compounds exhibit p*K*_i_ values greater than 7 and might show psychedelic properties at doses of a few tens of milligrams (Fig 4).

It may be noted that compound **38** was described by Nichols [19] (as **5a**) and found to be the most potent 5-HT_2A_ receptor agonist in his series of *N*-benzyl-5-methoxytryptamines, with *K*_i_ = 16.6 nM, EC_50_ = 1.9 nM and E_max_ = 81% (our values are 44.7 nM, 2.0 nM and 84%, respectively, in quite good agreement). It also gave an ED_50_ = 3.15 mg/kg in the mouse head twitch response (HTR) assay which is commonly viewed as a predictor of human psychedelic activity [25]. For the sake of comparison, the potent psychedelic 25I-NBOMe (4-iodo-2,5-dimethoxy-*N*-(2-methoxybenzyl)phenethylamine) exhibits a much lower *K*_i_ = 0.52 nM, almost identical IC_50_ and relative efficacy data, but its HTR result, ED_50_ = 0.078 mg/kg, suggests a 40-fold higher *in vivo* potency. Assuming that the HTR is a trustworthy model, we again see that the binding affinity seems to be a better predictor of psychedelic activity than functional potency, at least when determined as calcium mobilization.

A result that appeared with striking regularity was that almost all the compounds were partial agonists at the h5-HT_2A_ and full agonists at the h5-HT_2C_ receptor (or possibly “super agonists” eliciting a stronger response than serotonin). Moreover, a small number of these showed significant 5-HT_2C_ selectivity, sometimes coupled with *EC*_50_ values below 100 nM.

**Fig 5:**
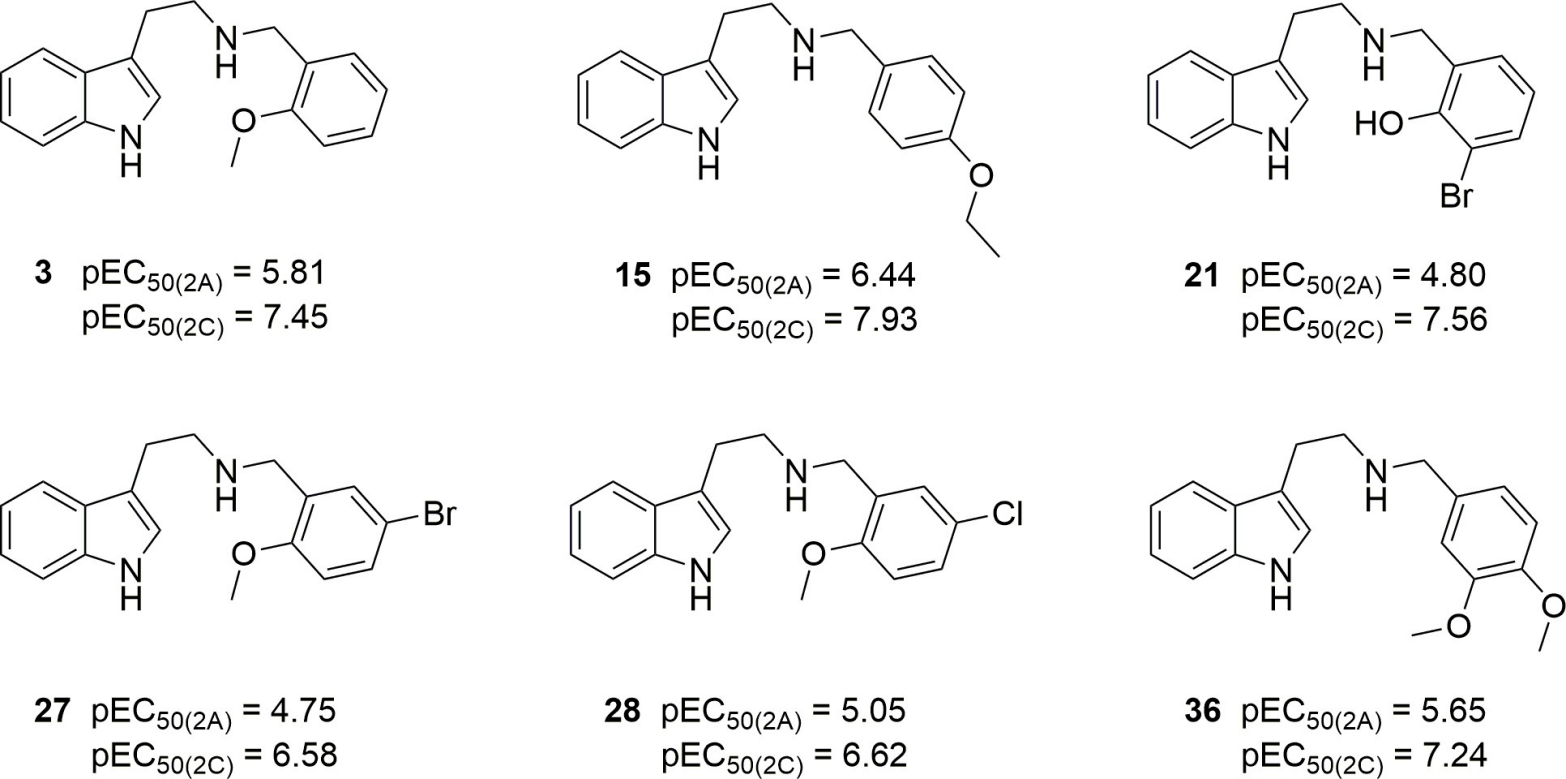
5-HT_2C_-selective *N*-benzyltryptamines.

*N-*(3-Bromo-2-hydroxybenzyl)tryptamine (**21**), which in spite of its modest 5-HT_2A_ affinity is an extremely weak h5-HT_2A_ partial agonist (p*EC*_50_ = 4.8) and a full agonist with a p*EC*_50_ of 7.6 (EC_50_ = 27 nM) at the h5-HT_2C_ receptor, is an extreme case that might be a particularly interesting candidate for *in vivo* studies. Other, less conspicuous examples, are the *N-*2-methoxybenzyl **3**, the *N-*4-ethoxybenzyl **15**, and the *N-*3,4-dimethoxybenzyl **36**. These compounds are 30 to 40-fold selective with *EC*_50_ values between 12 and 60 nM (p*EC*_50_ 7.95 to 7.24). Less potent, but still selective, are the 2’-methoxy-5´-halo derivatives **27** and **28** (Fig 5).

**Fig 6:**
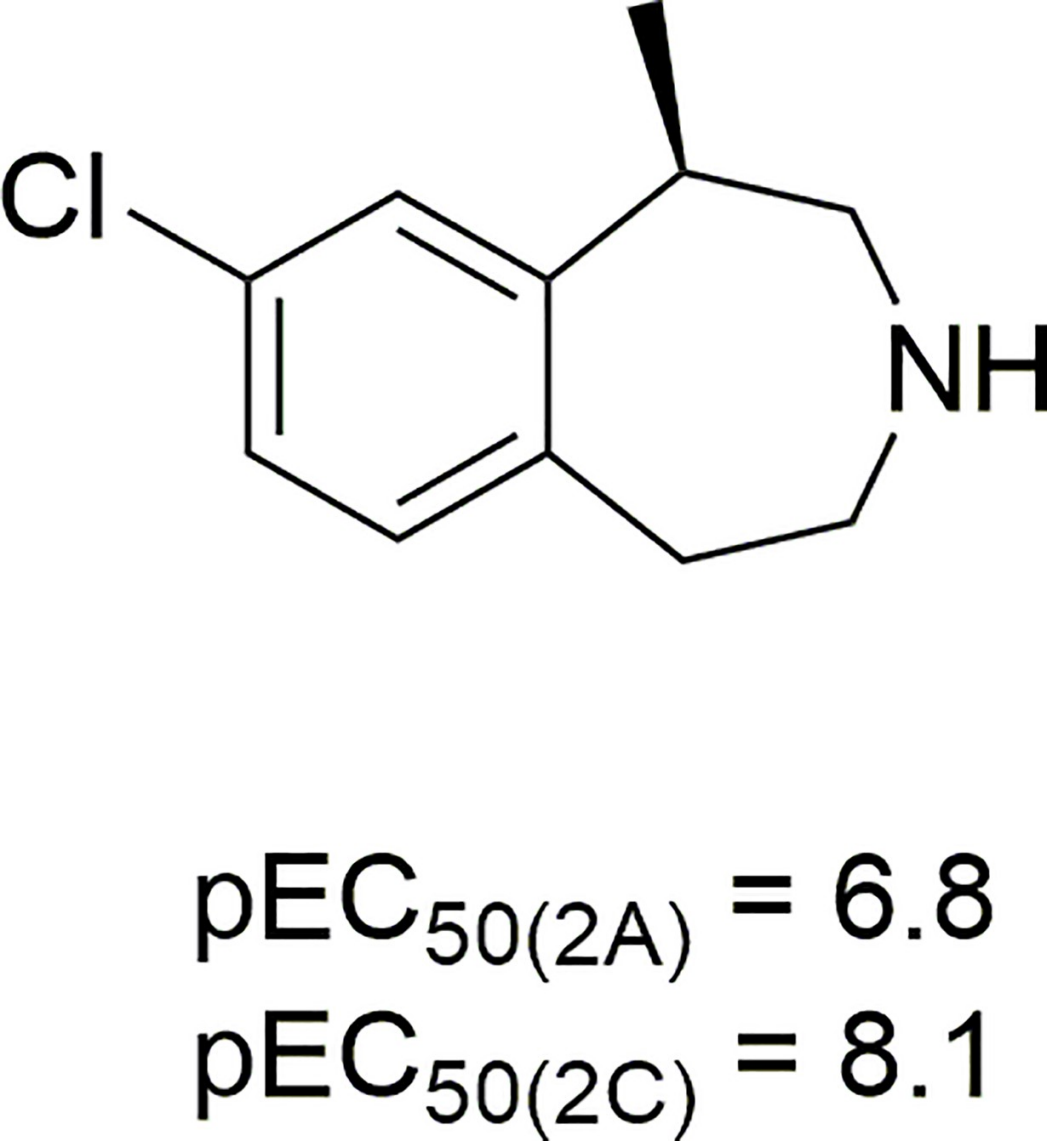
Structure and 5-HT_2A_ and 5-HT_2C_ functional potencies of lorcaserin.

Although not very potent, these *N*-benzylated tryptamines are more 5-HT_2C_/5-HT_2A_ selective than the approved 5-HT_2C_ agonist appetite suppressant lorcaserin (1(*R*)-8-chloro-1-methyl.2,3,4,5-tetrahydro-(1*H*)-3-benzazepine): *K*_i(2A/2C)_ about 8, and *EC*_50(2A/2C)_ about 20 [33] (Fig 6).

Lorcaserin is the first drug in its class to be approved by the FDA [34] as an anorexic. Like our *N*-benzylated tryptamine derivatives, lorcaserin is a full 5-HT_2C_ agonist, and a partial agonist (about 70% efficacy) at 5-HT_2A_ receptors [35]. Because of its modest 5-HT_2C_/5-HT_2A_ selectivity, lorcaserin is recommended exclusively for patients meeting specific criteria and has been placed in Schedule IV (prescription only) due to its presumed ability “to produce hallucinations, euphoria, and positive subjective responses at supratherapeutic doses” [34]. While **21** is about 3 times less potent than lorcaserin, it is much more selective vs. 5-HT_2A_ receptors, at which it also exhibits low efficacy, and therefore might not be expected to produce the abovementioned side effects at any reasonable dose level. Unfortunately, its appreciable affinity for the 5-HT_2B_ receptor militates against its acceptance, at least for prolonged use.

To summarize, our large series of *N*-benzylated tryptamines revealed a very broad range of affinities for the serotonin 5-HT_2_ receptor subtypes spread over three orders of magnitude and generally showing little selectivity (tenfold at most, but usually much less) between the 2A and 2C subtypes. The ability of these compounds to elicit calcium mobilization was also quite variable and with no obvious correlation with their affinity. Unlike the binding studies, the functional assays exhibited significant selectivity with an unexpected bias favoring the 5-HT_2C_ receptor. Besides, our compounds were generally full 2C agonists and only partial agonists, sometimes with rather low efficacy, at the 2A subtype. This fact, coupled with the selective activation of 5-HT_2C_ receptors by several of these substances points them out as possible leads for the development of a novel series of compounds of interest in the areas of appetite reduction and the treatment of drug abuse, schizophrenia and sexual dysfunctions.

## Materials and methods

### Chemistry

The tryptamine used was a generous gift from Prof. Michel Lebœuf, Faculté de Pharmacie de Châtenay-Malabry, Université de Paris XI, France. 5-Methoxytryptamine and most of the aldehydes were purchased from AK Scientific Inc. (Palo Alto, CA). Some aldehydes were prepared by standard aromatic bromination or *O*-methylation from the above precursors. Solvents were of synthesis grade, purchased from Merck S.A. (Santiago, Chile), and were used without additional purification. Melting points were determined on a Stuart SMP 10 apparatus, and NMR spectra were recorded on a Bruker Avance 400 instrument using the residual solvent signal as the internal standard.

The appropriate aromatic aldehyde (approx. 5 mmol) and the equivalent amount of tryptamine or 5-methoxytryptamine, each dissolved in MeOH (5 mL each) were placed in a 100 mL flask and allowed to react at r.t. for at least 120 min. Then solid NaBH_4_ (a small molar excess) was added in small portions with stirring, and the resulting solution was stirred at r.t. for another 12 h The MeOH was removed, the solid taken up in dilute HCl, the solution made alkaline and extracted with CH_2_Cl_2_. The organic extract was washed with water and dried over Na_2_SO_4_, the solvent was removed and the *N-*benzylated amine was purified by ball-to-ball distillation or column chromatography if necessary (Scheme 1).

The free bases were dissolved in the smallest possible volume of 2-PrOH, acetone, or MeOH, depending on their solubility. A small excess of 37% HCl diluted with 2-propanol was added, followed by Et_2_O (at least three volumes). The precipitated salt was filtered off, washed with Et_2_O and vacuum dried. Some hydroxyl-containing bases gave hygroscopic hydrochlorides, but their succinic or fumaric acid salts crystallized satisfactorily. To prepare them, the free bases were dissolved as above in acetone or MeOH, and treated dropwise, with stirring, with one equivalent or half an equivalent of a concentrated MeOH solution of succinic or fumaric acid to obtain the acid (hemisuccinate and hemifumarate) or the neutral (fumarate) salts. The resulting solutions were concentrated to dryness and the products recrystallized in acetone, CHCl_3_ or CH_2_Cl_2_, filtered, and vacuum dried.

***N-*Benzyl-[2-(1*H*-indol-3-yl)ethyl]amine (1) hydrochloride.** 75% yield, m.p. 188–189°C. ^1^H-NMR (400 MHz, DMSO-*d*_6_) δ = 10.96 (1H, s, NH-1), 9.33 (2H, brs, NH_2_^+^), 7.57 (3H, m, H3’, H5’, H7), 7.44 (3H, m, H2’, H4’, H6’) 7.36 (1H, dd, *J* ≈ 8 Hz, H4), 7.22 (1H, s, H2), 6.94–7.18 (2H, m, H5, H6), 4.19 (2H, unresolved t, α’-CH_2_), 3.13 (4H, brs, 2CH_2_).

***N-*(2-Hydroxybenzyl)-[2-(1*H*-indol-3-yl)ethyl]amine (2) hydrochloride.** 68% yield, m.p. 222–223°C. ^1^H-NMR (400 MHz, DMSO-*d*_6_) δ = 10.94 (1H, s, NH-1), 10.20 (1H, s, OH), 8.85 (2H, brs, NH_2_^+^), 7.53 (1H, dd, H7), 7.37 (2H, m, H4, H3’) 7.25 (1H, dd, H6’), 7.22 (1H, s, H2), 7.10 (1H, dd, H4ʼ), 6.96 (2H, m, H6, H5ʼ), 6.85 (1H, ddd, H5), 4.15 (2H, unresolved t, α’-CH_2_), 3.12 (4H, brs, 2CH_2_).

***N-*(2-Methoxybenzyl)-[2-(1*H*-indol-3-yl)ethyl]amine (3) hydrochloride.** 72% yield, m.p. 229–230°C. ^1^H-NMR (400 MHz, DMSO-*d*_6_) δ = 10.98 (1H, s, NH-1), 9.04 (2H, brs, NH_2_^+^), 7.55 (1H, dd, H7), 7.46 (2H, m, H3ʼ, H4ʼ), 7.39 (1H, dd, H4), 7.22 (1H, s, H2), 7.11 (2H, m, H6, H5ʼ), 7.00 (2H, m, H5, H6ʼ), 4.17 (2H, t, α’-CH_2_), 3.82 (3H, s, OCH_3_), 3.14 (4H, brs, 2CH_2_).

***N-*(2-Methylbenzyl)-[2-(1*H*-indol-3-yl)ethyl]amine (4) hydrochloride.** 78% yield. m.p. 209–210°C. ^1^H-NMR (400 MHz, DMSO-*d*_6_) δ = 10.99 (1H, s, NH-1), 9.41 (2H, brs, NH_2_^+^), 7.61 (1H, dd, H7), 7.57 (1H, dd, H6ʼ), 7.37 (1H, dd, H4), 7.30 (1H, ddd, H3ʼ), 7.27 (2H, m, H4ʼ, H5ʼ), 7.24 (1H, d, H2), 7.09 (1H, ddd, H6), 7.01 (1H, ddd, H5), 4.18 (2H, unresolved t, α’-CH_2_), 3.21 (4H, m, 2CH_2_), 2.40 (3H, s, CH_3_).

***N-*(2-Chlorobenzyl)-[2-(1*H*-indol-3-yl)ethyl]amine (5) hydrochloride.** 85% yield. m.p. 225–226°C. ^1^H-NMR (400 MHz, DMSO-*d*_6_) δ = 11.00 (1H, s, NH-1), 9.70 (2H, brs, NH_2_^+^), 7.84 (1H, dd, H3ʼ), 7.59 (1H, dd, H7), 7.55 (1H, ddd, H4ʼ), 7.45 (2H, m, H5ʼ, H6ʼ), 7.37 (1H, dd, H4), 7.24 (1H, s, H2), 7.09 (1H, ddd, H6), 7.00 (1H, ddd, H5), 4.32 (2H, unresolved t, α’-CH_2_), 3.20 (4H, m, 2CH_2_).

***N-*(2-Bromobenzyl)-[2-(1*H*-indol-3-yl)ethyl]amine (6) hydrochloride.** 87% yield. m.p. 228–229°C. ^1^H-NMR (400 MHz, DMSO-*d*_6_) δ = 10.98 (1H, s, NH-1), 9.51 (2H, brs, NH_2_^+^), 7.79 (1H, dd, H7), 7.73 (1H, dd, H3ʼ), 7.59 (1H, dd, H4), 7.50 (1H, td, H6ʼ), 7.38 (2H, m, H4ʼ, H5ʼ), 7.25 (1H, s, H2), 7.10 (1H, ddd, H6), 7.01 (1H, ddd, H5), 4.32 (2H, unresolved t, α’-CH_2_), 3.21 (4H, brs, 2CH_2_).

***N-*(3-Hydroxybenzyl)-[2-(1*H*-indol-3-yl)ethyl]amine (7) neutral fumarate.** 68% yield. m.p. 206–207°C. ^1^H-NMR (400 MHz, DMSO-*d*_6_) δ = 10.84 (1H, s, NH-1), 7.50 (1H, dd, H7), 7.33 (1H, dd, H4), 7.10 (1H, unresolved dd, H6ʼ), 6.82 (1H, unresolved d, H2ʼ), 6.80 (1H, dd, H5ʼ), 6.68 (1H, dd, H4ʼ), 7.15 (1H, s, H2), 7.06 (1H, ddd, H6), 6.96 (1H, ddd, H5), 3.83 (2H, unresolved t, α’-CH2), 2.93 (4H, brs, 2CH_2_), 6.47 (1H, s, fumarate).

***N-*(3-Methylbenzyl)-[2-(1*H*-indol-3-yl)ethyl]amine (8) hydrochloride.** 85% yield. m.p. 189–190°C. ^1^H-NMR (400 MHz, DMSO-*d*_6_) δ = 10.98 (1H, s, NH-1), 9.43 (2H, brs, NH2+) 7.56 (1H, dd, H7), 7.37 (3H, m, H4ʼ, H5ʼ, H6ʼ), 7.34 (1H, dd, H4), 7.26 (1H, d, H2ʼ), 7.22 (1H, s, H2), 7.09 (1H, ddd, H6), 6.99 (1H, ddd, H5), 4.14 (2H, unresolved t, α’-CH_2_), 3.13 (4H, brs, 2CH_2_), 2.32 (3H, s, CH_3_).

***N-*(3-Fluorobenzyl)-[2-(1*H*-indol-3-yl)ethyl]amine (9) hydrochloride.** 86% yield. m.p. 223–225°C. ^1^H-NMR (400 MHz, DMSO-*d*_6_) δ = 10.97 (1H, s, NH-1), 9.58 (2H, brs, NH_2_^+^) 7.57 (1H, dd, H7), 7.55 (1H, ddd, H4ʼ), 7.49 (1H, unresolved dd, H5ʼ), 7.44 (1H, unresolved d, H2ʼ), 7.36 (1H, dd, H4), 7.27 (1H, unresolved dd, H6ʼ), 7.23 (1H, s, H2), 7.09 (1H, ddd, H6), 7.00 (1H, ddd, H5), 4.21 (2H, unresolved t, α’-CH_2_), 3.14 (4H, brs, 2CH_2_).

***N-*(3-Chlorobenzyl)-[2-(1*H*-indol-3-yl)ethyl]amine (10) hydrochloride.** 83% yield. m.p. 209–211°C. ^1^H-NMR (400 MHz, DMSO-*d*_6_) δ = 10.98 (1H, s, NH-1), 9.62 (2H, brs, NH_2_^+^) 7.74 (1H, s, H2ʼ), 7.58 (2H, m, H7, H4ʼ), 7.47 (2H, unresolved signals, H5ʼ, H6ʼ), 7.36 (1H, dd, H4), 7.22 (1H, s, H2), 7.09 (1H, ddd, H6), 7.00 (1H, ddd, H5), 4.20 (2H, unresolved t, α’-CH_2_), 3.14 (4H, brs, 2CH_2_).

***N-*(3-Bromobenzyl)-[2-(1*H*-indol-3-yl)ethyl]amine (11) hydrochloride.** 88% yield. m.p. 216–218°C. ^1^H-NMR (400 MHz, DMSO-*d*_6_) δ = 10.98 (1H, s, NH-1), 9.54 (2H, brs, NH2+) 7.86 (1H, s, H2ʼ), 7.59 (3H, m, H7, H4ʼ, H5ʼ), 7.40 (2H, unresolved signals, H4, H6ʼ), 7.22 (1H, s, H2), 7.10 (1H, ddd, H6), 6.97 (1H, ddd, H5), 4.20 (2H, unresolved t, α’-CH2), 3.14 (4H, brs, 2CH_2_).

***N-*(4-Hydroxybenzyl)-[2-(1*H*-indol-3-yl)ethyl]amine (12) (free base).** 95% yield. m.p. n.d. ^1^H-NMR (400 MHz, DMSO-*d*_6_) δ = 10.76 (1H, s, NH-1), 7.48 (1H, dd, H7), 7.32 (1H, dd, H4), 7.09 (1H, s, H2), 7.08 (2H, d, H2ʼ, H6ʼ), 7.05 (1H, ddd, H6), 6.95 (1H, ddd, H5), 6.68 (2H, d, H3ʼ, H5ʼ), 3.61 (2H, s, α’-CH2), 2.84 (2H, t, CH2), 2.76 (2H, t, CH_2_).

***N-*(4-Methoxybenzyl)-[2-(1*H*-indol-3-yl)ethyl]amine (13) hydrochloride.** 78% yield. m.p. 199–200°C. ^1^H-NMR (400 MHz, DMSO-*d*_6_) δ = 10.98 (1H, s, NH-1), 9.26 (2H, brs, NH2+), 7.55 (1H, dd, H7), 7.49 (2H, d, H2ʼ, H6ʼ), 7.36 (1H, dd, H4), 7.22 (1H, s, H2), 7.09 (1H, ddd, H6), 6.99 (3H, m, H5, H3ʼ, H5ʼ), 4.11 (2H, unresolved t, α’-CH_2_), 3.77 (3H, s, OCH_3_), 3.10 (4H, brs, 2CH_2_).

***N-*(4-Methylbenzyl)-[2-(1*H*-indol-3-yl)ethyl]amine (14) hydrochloride.** 73% yield. m.p. 213–214°C. ^1^H-NMR (400 MHz, DMSO-d6) δ = 10.97 (1H, s, NH-1), 9.41 (2H, brs, NH2+), 7.56 (1H, dd, H7), 7.46 (2H, d, H2ʼ, H6ʼ), 7.36 (1H, dd, H4), 7.23 (2H, d, H3ʼ, H5ʼ), 7.21 (1H, s, H2), 7.09 (1H, ddd, H6), 6.98 (1H, ddd, H5), 4.13 (2H, unresolved t, α’-CH_2_), 3.12 (4H, brs, 2CH_2_), 2.32 (3H, s, CH_3_).

***N-*(4-Ethoxybenzyl)-[2-(1*H*-indol-3-yl)ethyl]amine (15) hydrochloride.** 82% yield. m.p. 206–208°C. ^1^H-NMR (400 MHz, DMSO-*d*_6_) δ = 10.98 (1H, s, NH-1), 9.38 (2H, brs, NH2+), 7.56 (1H, dd, H7), 7.49 (2H, d, H2ʼ, H6ʼ), 7.36 (1H, dd, H4), 7.21 (1H, s, H2), 7.09 (1H, ddd, H6), 6.98 (3H, m, H5, H3ʼ, H5ʼ), 4.07 (2H, unresolved t, α’-CH2), 4.00 (2H, unresolved quadruplet, ethoxyl CH2) 3.11 (4H, brs, 2CH2), 1.32 (3H, t, ethoxyl CH_3_).

***N-*(4-Chlorobenzyl)-[2-(1*H*-indol-3-yl)ethyl]amine (16) hydrochloride.** 85% yield. m.p. 229–230°C. ^1^H-NMR (400 MHz, DMSO-*d*_6_) δ = 10.97 (1H, s, NH-1), 9.52 (2H, brs, NH2+), 7.62 (2H, overlapping d, H3ʼ, H5ʼ), 7.58 (1H, overlapping dd, H7), 7.50 (2H, overlapping d, H2ʼ, H6ʼ), 7.36 (1H, dd, H4), 7.22 (1H, s, H2), 7.09 (1H, ddd, H6), 6.99 (1H, ddd, H5), 4.19 (2H, unresolved t, α’-CH_2_), 3.13 (4H, brs, 2CH_2_).

***N-*(4-Bromobenzyl)-[2-(1*H*-indol-3-yl)ethyl]amine (17) hydrochloride.** 86% yield. m.p. 238–240°C. ^1^H-NMR (400 MHz, DMSO-*d*_6_) δ = 10.96 (1H, s, NH-1), 9.43 (2H, brs, NH2+), 7.65 (2H, d, H3ʼ, H5ʼ), 7.55 (3H, m, H2ʼ, H6ʼ, H7), 7.36 (1H, dd, H4), 7.22 (1H, s, H2), 7.09 (1H, ddd, H6), 7.00 (1H, ddd, H5), 4.17 (2H, unresolved t, α’-CH_2_), 3.13 (4H, brs, 2CH_2_).

***N-*(4-Nitrobenzyl)-[2-(1*H*-indol-3-yl)ethyl]amine (18) hydrochloride.** 90% yield. m.p. 212–214°C. ^1^H-NMR (400 MHz, DMSO-*d*_6_) δ = 10.99 (1H, s, NH-1), 9.80 (2H, brs, NH2+), 8.28 (2H, d, H3ʼ, H5ʼ), 7.89 (2H, d, H2ʼ, H6ʼ), 7.59 (1H, dd, H7), 7.36 (1H, dd, H4), 7.23 (1H, s, H2), 7.09 (1H, ddd, H6), 7.00 (1H, ddd, H5), 4.35 (2H, unresolved t, α’-CH_2_), 3.17 (4H, brs, 2CH_2_).

***N-*(2-Hydroxy-3-methoxybenzyl)-[2-(1*H*-indol-3-yl)ethyl]amine (19) neutral succinate.** 58% yield. m.p. 109–111°C. ^1^H-NMR (400 MHz, DMSO-d6) δ = 10.83 (1H, s, NH-1), 7.49 (1H, dd, H7), 7.33 (1H, dd, H4), 7.15 (1H, s, H2), 7.06 (1H, ddd, H6), 6.97 (1H, ddd, H5), 6.86 (1H, dd, H5ʼ), 6.71 (2H, overlapping dd, H4ʼ, H6ʼ), 3.94 (2H, unresolved t, α’-CH_2_), 3.75 (3H, s, OCH_3_), 2.91 (4H, brs, 2CH_2_), 2.32 (2H, s, succinate).

***N-*(2,3-Dimethoxybenzyl)-[2-(1*H*-indol-3-yl)ethyl]amine (20) hydrochloride.** 70% yield. m.p. 210–212°C. ^1^H-NMR (400 MHz, DMSO-*d*_6_) δ = 10.99 (1H, s, NH-1), 9.35 (2H, brs, NH2+), 7.56 (1H, dd, H7), 7.36 (1H, dd, H4), 7.22 (1H, s, H2), 7.20 (1H, overlapping dd, H5ʼ), 7.12 (2H, overlapping dd, H4ʼ, H6ʼ), 7.09 (1H, overlapping ddd, H6), 6.99 (1H, ddd, H5), 4.17 (2H, unresolved t, α’-CH2), 3.83 (3H, s, OCH3-3ʼ), 3.79 (3H, s, OCH3-2ʼ), 3.14 (4H, brs, 2CH2).

***N-*(3-Bromo-2-hydroxybenzyl)-[2-(1*H*-indol-3-yl)ethyl]amine (21) neutral succinate.** 75% yield. m.p. 141–142°C. ^1^H-NMR (400 MHz, DMSO-d6) δ = 10.83 (1H, s, NH-1), 7.50 (1H, dd, H7), 7.37 (1H, dd, H4ʼ), 7.33 (1H, dd, H4), 7.17 (1H, s, H2), 7.05 (2H, overlapping ddd, H6, H6ʼ), 6.96 (1H, ddd, H5), 6.64 (1H, t, H5ʼ), 3.99 (2H, unresolved t, α’-CH_2_), 2.89 (4H, brs, 2CH_2_), 2.39 (2H, s, succinate).

***N-*(3-Fluoro-2-hydroxybenzyl)-[2-(1*H*-indol-3-yl)ethyl]amine (22) neutral succinate.** 72% yield. m.p. 151–152°C. ^1^H-NMR (400 MHz, DMSO-d6) δ = 10.82 (1H, s, NH-1), 7.50 (1H, dd, H7), 7.33 (1H, dd, H4), 7.16 (1H, s, H2), 7.06 (2H, overlapping ddd, H6, H4ʼ), 6.97 (1H, ddd, H5), 6.91 (1H, dd, H5ʼ), 6.70 (1H, quadruplet, H6ʼ), 3.99 (2H, unresolved t, α’-CH_2_), 2.90 (4H, brs, 2CH_2_), 2.35 (2H, s, succinate).

***N-*(2-Hydroxy-5-methylbenzyl)-[2-(1*H*-indol**-3-yl)ethyl]amine (23) acid fumarate. 48% yield. m.p. 183–185°C. 1H-NMR (400 MHz, DMSO-d6) δ = 10.89 (1H, s, NH-1), 7.51 (1H, dd, H7), 7.35 (1H, dd, H4), 7.17 (1H, s, H2), 7.07 (1H, ddd, H6), 7.01 (1H, d, H6ʼ), 6.97 (1H, overlapping ddd, H5), 6.93 (1H, overlapping dd, H4ʼ), 6.70 (1H, d, H3ʼ), 3.95 (2H, unresolved t, α’-CH_2_), 2.96 (4H, brs, 2CH_2_), 2.17 (3H, s, CH_3_-5ʼ), 6.49 (2H, s, fumarate).

***N-*(5-Fluoro-2-hydroxybenzyl)-[2-(1*H*-indol-3-yl)ethyl]amine (24) hydrochloride.** 62% yield. m.p. 185–186°C. ^1^H-NMR (400 MHz, DMSO-d6) δ = 10.82 (1H, s, NH-1), 7.50 (1H, dd, H7), 7.33 (1H, dd, H4), 7.16 (1H, s, H2), 7.07 (1H, ddd, H6), 7.01 (1H, dd, H3ʼ), 6.98 (1H, ddd, H5), 6.91 (1H, ddd, H4ʼ), 6.72 (1H, dd, H6ʼ), 3.90 (2H, s, α’-CH2), 2.90 (4H, brs, 2CH_2_).

***N-*(5-Fluoro-2-methoxybenzyl)-[2-(1*H*-indol-3-yl)ethyl]amine (25) hydrochloride.** 70% yield. m.p. 179–180°C. ^1^H-NMR (400 MHz, DMSO-d6) δ = 10.99 (1H, s, NH-1), 9.32 (2H, brs, NH2+), 7.55 (1H, dd, H7), 7.47 (1H, dd, H4ʼ), 7.36 (1H, dd, H4), 7.23 (2H, m, H2, H6ʼ), 7.09 (2H, m, H6, H3ʼ), 6.99 (1H, ddd, H5), 4.15 (2H, unresolved t, α’-CH_2_), 3.80 (3H, s, OCH_3_), 3.14 (4H, brs, 2CH_2_).

***N-*(5-Bromo-2-hydroxybenzyl)-[2-(1*H*-indol-3-yl)ethyl]amine (26) hydrochloride.** 45% yield. m.p. 188–189°C. ^1^H-NMR (400 MHz, DMSO-*d*_6_) δ = 10.95 (1H, s, NH-1), 7.53 (1H, dd, H7), 7.47 (1H, d, H3ʼ), 7.32 (1H, dd, H4), 7.13 (2H, m, H6, H4ʼ), 7.05 (1H, ddd, H5), 6.96 (1H, s H2,), 5.98 (1H, s, H6ʼ), 3.43 (2H, unresolved t, α’-CH2), 3.07 (4H, m, 2CH_2_).

***N-*(5-Bromo-2-methoxybenzyl)-[2-(1*H*-indol-3**-yl)ethyl]amine (27) hydrochloride. 50% yield. m.p. 192–193°C. 1H-NMR (400 MHz, DMSO-d6) δ = 11.00 (1H, s, NH-1), 9.29 (2H, brs, NH2+), 7.76 (1H, d, H6ʼ), 7.57 (2H, overlapping dd, H7, H4ʼ), 7.37 (1H, dd, H4), 7.23 (1H, s, H2), 7.09 (2H, m, H6, H3ʼ), 7.00 (1H, ddd, H5), 4.15 (2H, t, α’-CH_2_), 3.81 (3H, s, OCH_3_), 3.14 (4H, brs, 2CH_2_).

***N-*(5-Chloro-2-methoxybenzyl)-[2-(1*H*-indol-3-yl)ethyl]amine (28) hydrochloride.** 72% yield. m.p. 184–185°C. ^1^H-NMR (400 MHz, DMSO-*d*_6_) δ = 11.00 (1H, s, NH-1), 9.29 (2H, brs, NH2+), 7.65 (1H, d, H6ʼ), 7.57 (1H, dd, H7), 7.46 (1H, dd, H4ʼ), 7.37 (1H, dd, H4), 7.23 (1H, s, H2), 7.09 (2H, m, H6, H3ʼ), 7.00 (1H, ddd, H5), 4.15 (2H, unresolved t, α’-CH_2_), 3.82 (3H, s, OCH_3_), 3.15 (4H, brs, 2CH_2_).

***N-*(2,5-Dimethoxybenzyl)-[2-(1*H*-indol-3-yl)ethyl]amine (29) hydrochloride.** 86% yield. m.p. 175–176°C. ^1^H-NMR (400 MHz, DMSO-*d*_6_) δ = 10.99 (1H, s, NH-1), 9.22 (2H, brs, NH_2_^+^), 7.55 (1H, dd, H7), 7.36 (1H, dd, H4), 7.22 (1H, s, H2), 7.21 (1H, overlapping d, H6ʼ), 7.09 (1H, ddd, H6,), 6.99 (2H, overlapping dd, H3ʼ, H4ʼ), 6.95 (1H, ddd, H5), 4.13 (2H, s, α’-CH2), 3.76 (3H, s, OCH3-2ʼ), 3.73 (3H, s, OCH3-5ʼ), 3.13 (4H, brs, 2CH_2_).

***N-*(5-Nitro-2-hydroxybenzyl)-[2-(1*H*-indol-3-yl)ethyl]amine (30) hydrochloride.** 76% yield. m.p. 194–196°C. 1H-NMR (400 MHz, DMSO-d6) δ = 10.95 (1H, s, NH-1), 8.00 (1H, s, H6ʼ), 7.88 (1H, dd, H4ʼ), 7.53 (1H, dd, H7), 7.35 (1H, dd, H4), 7.22 (1H, s, H2), 7.08 (1H, ddd, H6,), 6.98 (1H, ddd, H5), 6.29 (1H, dd, H3ʼ), 4.02 (2H, unresolved t, α’-CH_2_), 3.05 (4H, brs, 2CH_2_).

***N-*(4-Bromo-2-hydroxybenzyl)-[2-(1*H*-indol-3-yl)ethyl]amine (31) hydrochloride.** 66% yield. m.p. 192–194°C. 1H-NMR (400 MHz, DMSO-d6) δ = 10.81 (1H, s, NH-1), 8.99 (2H, brs, NH2+), 7.54 (1H, dd, H7), 7.36 (2H, overlapping dd, H4, H5ʼ), 7.22 (1H, s, H2), 7.16 (1H, d, H3ʼ), 7.09 (1H, overlapping ddd, H6), 7.04 (1H, overlapping dd, H6ʼ), 7.00 (1H, overlapping dd, H5), 4.11 (2H, unresolved t, α’-CH_2_), 3.12 (4H, brs, 2CH_2_).

***N-*(2,4-Dimethoxybenzyl)-[2-(1*H*-indol-3-yl)ethyl]amine (32) hydrochloride.** 73% yield. m.p. 184–186°C. ^1^H-NMR (400 MHz, DMSO-*d*_6_) δ = 10.99 (1H, s, NH-1), 9.03 (2H, brs, NH2+), 7.54 (1H, dd, H7), 7.38 (2H, overlapping dd, H4, H5ʼ), 7.22 (1H, s, H2), 7.09 (1H, overlapping ddd, H6), 6.99 (1H, overlapping ddd, H5), 6.61 (1H, d, H3ʼ), 6.57 (1H, d, H6ʼ), 4.07 (2H, unresolved t, α’-CH2), 3.80 (3H, s, OCH3-2ʼ), 3.78 (3H, s, OCH3-4ʼ), 3.11 (4H, brs, 2CH2).

***N-*(6-Bromo-2-hydroxybenzyl)-[2-(1*H*-indol-3-yl)ethyl]amine (33) hydrochloride.** 62% yield. m.p. 195–196°C. ^1^H-NMR (400 MHz, DMSO-*d*_6_) δ = 11.07 (1H, s, NH-1), 11.01 (1H, s, OH), 9.09 (2H, brs, NH_2_^+^), 7.55 (1H, dd, H7), 7.36 (1H, dd, H4), 7.23 (1H, s, H2), 7.19 (1H, dd, H5ʼ), 7.13 (1H, dd, H4ʼ), 7.08 (1H, ddd, H6), 7.07 (1H, dd, H3ʼ), 7.00 (1H, ddd, H5), 4.29 (2H, s, α’-CH2), 3.17 (4H, brs, 2CH2).

***N-*(6-Fluoro-2-hydroxybenzyl)-[2-(1*H*-indol-3-yl)ethyl]amine (34) neutral succinate.** 58% yield. m.p. 159–161°C. ^1^H-NMR (400 MHz, DMSO-d6) δ = 10.82 (1H, s, NH-1), 7.49 (1H, dd, H7), 7.33 (1H, dd, H4), 7.15 (1H, s, H2), 7.11 (1H, dd, H5ʼ), 7.06 (1H, ddd, H6), 6.97 (1H, ddd, H5), 6.58 (2H, overlapping dd, H3ʼ, H4ʼ), 3.97 (2H, unresolved t, α’-CH_2_), 2.90 (4H, brs, 2CH_2_), 2.35 (2H, s, succinate).

***N-*(3,5-Dibromo-2-hydroxybenzyl)-[2-(1*H*-indol-3-yl)ethyl]amine (35) hydrochloride.** 65% yield. m.p. 183–184°C. 1H-NMR (400 MHz, DMSO-d6) δ = 10.97 (1H, s, NH-1), 7.82 (1H, d, H4ʼ), 7.71 (1H, d, H6ʼ), 7.57 (1H, dd, H7), 7.38 (1H, dd, H4), 7.24 (1H, s, H2), 7.10 (1H, ddd, H6), 7.02 (1H, ddd, H5), 4.23 (2H, unresolved t, α’-CH_2_), 3.18 (2H, unresolved t, CH_2_), 3.11 (2H, unresolved t, CH_2_).

***N-*(3,4-Dimethoxybenzyl)-[2-(1*H*-indol-3-yl)ethyl]amine (36) hydrochloride.** 83% yield. m.p. 238–240°C. ^1^H-NMR (400 MHz, DMSO-*d*_6_) δ = 10.97 (1H, s, NH-1), 9.34 (2H, brs, NH2+), 7.56 (1H, dd, H7), 7.36 (1H, dd, H4), 7.31 (1H, dd, H2ʼ), 7.22 (1H, s, H2), 7.08 (1H, overlapped d, H6ʼ), 7.06 (1H, overlapping ddd, H6), 7.00 (1H, d, H5ʼ), 6.97 (1H, overlapped ddd, H5), 4.10 (2H, unresolved t, α’-CH2), 3.78 (3H, s, OCH3-3ʼ), 3.76 (3H, s, OCH3-4ʼ), 3.12 (4H, brs, 2CH2).

***N-*Benzyl-[2-(5-methoxy-1*H*-indol-3-yl)ethyl]amine (37) hydrochloride.** 84% yield. m.p. 233–234°C. ^1^H-NMR (400 MHz, DMSO-d6) δ = 10.80 (1H, s, NH-1), 9.51 (2H, brs, NH2+), 7.60 (2H, m, H2ʼ, H6ʼ), 7.42 (3H, m, H3ʼ, H4ʼ, H5ʼ), 7.24 (1H, d, H7), 7.16 (1H, s, H2), 7.10 (1H, d, H4), 6.73 (1H, dd, H6), 4.18 (2H, t, α’-CH_2_), 3.76 (3H, s, OCH_3_-5), 3.10 (4H, brs, 2CH_2_).

***N-*(2-Methoxybenzyl)-[2-(5-methoxy-1*H*-indol-3-il)ethyl]amine (38) hydrochloride.** 71% yield. m.p. 240–241°C. ^1^H-NMR (400 MHz, DMSO-d6) δ = 10.81 (1H, s, NH-1), 8.79 (2H, brs, NH2+), 7.49 (1H, dd, H6ʼ), 7.39 (1H, ddd, H5ʼ), 7.25 (1H, d, H7), 7.16 (1H, s, H2), 7.06 (2H, m, H4, H3ʼ), 6.98 (1H, ddd, H4ʼ), 6.74 (1H, dd, H6), 4.10 (2H, s, α’-CH2), 3.79 (3H, s, OCH3-2ʼ), 3.75 (3H, s, OCH3-5), 3.07 (4H, brs, 2CH2).

***N-*(2-**Chlorobenzyl)-[2-(5-methoxy-1H-indol-3-yl)ethyl]amine (39) hydrochloride. 85% yield. m.p. 193–194°C. 1H-NMR (400 MHz, DMSO-d6) δ = 10.82 (1H, s, NH-1), 9.64 (2H, brs, NH2+), 7.83 (1H, ddd, H4ʼ), 7.56 (1H, ddd, H5ʼ), 7.45 (2H, overlapped dd, H3ʼ, H6ʼ), 7.25 (1H, d, H7), 7.19 (1H, d, H2), 7.12 (1H, s, H4), 6.75 (1H, dd, H6), 4.32 (2H, s, α’-CH_2_), 3.77 (3H, s, OCH_3_-5), 3.17 (4H, m, 2CH_2_).

***N-*(2-Bromobenzyl)-[2-(5-methoxy-1*H*-indol-3-yl)ethyl]amine (40) hydrochloride.** 83% yield. m.p. 206–207°C. ^1^H-NMR (400 MHz, DMSO-d6) δ = 10.79 (1H, s, NH-1), 9.56 (2H, brs, NH2+), 7.80 (1H, dd, H3ʼ), 7.73 (1H, dd, H6ʼ), 7.50 (1H, ddd, H4ʼ), 7.37 (1H, ddd, H5ʼ), 7.26 (1H, d, H7), 7.20 (1H, s, H2), 7.11 (1H, d, H4), 6.75 (1H, dd, H6), 4.32 (2H, s, α’-CH_2_), 3.77 (3H, s, OCH_3_-5), 3.17 (4H, m, 2CH_2_).

***N-*(4-Bromobenzyl)-[2-(5-methoxy-1*H*-indol-3-yl)ethyl]amine (41) hydrochloride.** 80% yield. m.p. 240–241°C. ^1^H-NMR (400 MHz, DMSO-*d*_6_) δ = 10.80 (1H, s, NH-1), 9.36 (2H, brs, NH2+), 7.66 (2H, dd, H3ʼ, H5ʼ), 7.53 (2H, dd, H2ʼ, H6ʼ), 7.25 (1H, d, H7), 7.17 (1H, s, H2), 7.06 (1H, d, H4), 6.74 (1H, dd, H6), 4.18 (2H, s, α’-CH_2_), 3.76 (3H, s, OCH_3_-5), 3.09 (4H, brs, 2CH_2_).

***N-*(2-Hydroxy-5-methoxybenzyl)-[2-(5-methoxy-1*H*-indol-3-yl)ethyl]amine (42) acid fumarate.** 48% yield. m.p. 196–197°C. ^1^H-NMR (400 MHz, DMSO-d6) δ = 10.74 (1H, s, NH-1), 7,22 (1H, d, H7), 7.13 (1H, s, H2), 7.00 (1H, d, H4), 6.92 (1H, d, H6ʼ), 6.78 (2H, m, H3ʼ, H4ʼ), 6.72 (1H, dd, H6), 6.53 (2H, s, fumarate), 4.02 (2H, s, α’-CH2), 3.74 (3H, s, OCH3-5ʼ), 3.66 (3H, s, OCH3-5), 3.00 (4H, brs, 2CH).

***N-*(5-Fluoro-2-hydroxybenzyl)-[2-(5-methoxy-1*H*-indol-3-yl)ethyl]amine (43) neutral fumarate.** 55% yield. m.p. 200–201°C. ^1^H-NMR (400 MHz, DMSO-d6) δ = 10.66 (1H, s, NH-1), 7.22 (1H, d, H7), 7.11 (1H, s, H2), 7.04 (1H, dd, H4ʼ), 6.97 (1H, d, H4), 6.90 (1H, d, H3ʼ), 6.73 (2H, m, H6, H6ʼ), 6.51 (1H, s, fumarate), 3.92 (2H, unresolved t, α’-CH2), 3.74 (3H, s, OCH3-5), 2.89 (4H, brs, 2CH2).

## Pharmacology

### Binding studies

#### Competition binding to the human 5-HT_2A_ receptor

Serotonin 5-HT_2A_ receptor competition binding experiments were carried out in polypropylene 96-well plates. In each well were incubated 60 μg of membranes from a CHO-5-HT_2A_ cell line prepared in our laboratory, 1 nM [^3^H]ketanserin (47.3 Ci/mmol, 1 mCi/ml, Perkin Elmer NET791250UC), studied compounds and standard. Non-specific binding was determined in the presence of methysergide 1 μM (Sigma M137). The reaction mixture (Vt: 250 μL/well) was incubated at 37°C for 30 min, 200 μL was transferred to a GF/B 96-well plate (Millipore, Madrid, Spain) pretreated with 0.5% of PEI and treated with binding buffer (Tris-HCl 50 mM, pH = 7.4), and was filtered and washed six times with 250 μL wash buffer (Tris-HCl 50 mM, pH = 6.6), and 35 μL of Universol Scintillation cocktail (Perkin Elmer, Alcobendas, Spain) were added to each well before counting in a microplate beta scintillation counter (Microbeta Trilux, PerkinElmer, Madrid, Spain).

#### Competition binding to the human 5-HT_2B_ receptor

Serotonin 5-HT_2B_ receptor competition binding experiments were carried out in polypropylene 96-well plates. In each well were incubated 5 μg of membranes from a CHO-5-HT_2B_ cell line prepared in our laboratory, 1 nM [^3^H]LSD (75.9 Ci/mmol, 1 mCi/ml, Perkin Elmer NET638250UC), studied compounds and standard. Non-specific binding was determined in the presence of 5-HT 50 μM (Sigma H9523). The reaction mixture (Vt: 250 μL/well) was incubated at 37°C for 30 min, 200 μL was transferred to a GF/C 96-well plate (Millipore, Madrid, Spain) pretreated with 0.5% of PEI and treated with binding buffer (Tris-HCl 50 mM, ascorbic acid 0.1%, CaCl_2_ 4 mM, pH = 7.4), and was filtered and washed four times with 250 μL wash buffer (Tris-HCl 50 mM, pH = 7.4), and 35 μL of Universol Scintillation cocktail (Perkin Elmer, Alcobendas, Spain) were added to each well before counting in a microplate beta scintillation counter (Microbeta Trilux, PerkinElmer, Madrid, Spain).

#### Competition binding to the human 5-HT_2C_ receptor

Serotonin 5-HT_2C_ receptor competition binding experiments were carried out in polypropylene 96-well plates. In each well were incubated 3 μg of membranes from a Hela-5-HT_2C_ cell line prepared in our laboratory, 1.25 nM [^3^H]mesulergine (83.1 Ci/mmol, 1 mCi/ml, Perkin Elmer NET1148250UC), studied compounds and standard. Non-specific binding was determined in the presence of mianserin 10 μM (Sigma M2525). The reaction mixture (Vt: 250 μL/well) was incubated at 27°C for 60 min, 200 μL was transferred to a GF/C 96-well plate (Millipore, Madrid, Spain) pretreated with 0.5% of PEI and treated with binding buffer (Tris-HCl 50 mM, pH = 7.5), and was filtered and washed four times with 250 μL wash buffer (Tris-HCl 50 mM, pH = 6.6), and 35 μL of Universol Scintillation cocktail (Perkin Elmer, Alcobendas, Spain) were added to each well before counting in a microplate beta scintillation counter (Microbeta Trilux, PerkinElmer, Madrid, Spain).

### Functional studies

Functional activities were assessed by measuring Ca^2+^ release in CHO-5-HT_2A_ or HeLa-5-HT_2C_ cells. The day before the assay, 2000 (5-HT_2A_) or 10000 (5-HT_2C_) cells/well were seeded in 384 well black plates (Greiner 781091). The cells were incubated with 25 μL of Fura-2 QBTTM Calcium Kit (Molecular Devices), in buffer supplemented with 5 mM probenecid (Invitrogen) for 1 h at 37°C. Changes in fluorescence due to intracellular Ca^2+^ mobilization (λ_ex_ = 340 nm, λ_ex_ = 380 nm; λ_em_ = 540 nm) were measured using a calcium imaging plate reader system (FDSS7000EX, Hamamatsu) every second after the establishment of a baseline. The agonist Ca^2+^ peak in response to agonist addition occurred from 10 to 20 s following stimulation.

### Statistics

Data were adjusted to non-linear fitting using Prism V2.1 software (Graph Pad Inc., Chicago, USA). K_i_ values were calculated using the Cheng-Prusoff equation.
